# Diabetic Coma

**Published:** 1891-02-07

**Authors:** 


### DIABETIC COMA.

Dr. Schmitz, in the *Berlin. Med. Wochensch.*, insists on the practical importance of distinguishing between the two forms of coma—due respectively to cardiac failure and to auto-intoxication. To the latter it is at present the fashion to apply the name of acetonæmia, but he believes it to be caused by the absorption of products of fæcal decomposition, the precise nature of which is as yet unknown. Cardiac failure is the result of the general destruction of the striped muscular tissue caused by the presence of sugar in the blood, and indicated by the extreme want of tone and weakness of the voluntary muscles with consequent fatigue and loss of power. The participation of the heart in these degenerative changes is seen in the small, slow pulse, accelerated on the least exertion, feeble apex beat, and weakened first sound. There are dyspnœa, cyanosis, and ultimately coma from poisoning by the excess of carbonic acid in the blood. The treatment of this condition consists, of course, in rest and quietude, light nutritious diet, and the cautious use of stimulants such as alcohol, and more freely of ammonia, camphor, and ether. The patient must be kept in the horizontal position, and strong coffee may be given with advantage. If he recover, absolute rest should be enforced until the first sound has again become normal. The other condition, the so-called acetonæmia, is preceded, according to Dr. Schmitz, by loss of appetite, fatigue and somnolence, obstinate constipation, dry furred tongue, foul breath, and ill-smelling eructations. There are often colicky pains, and sometimes a greenish fluid is vomited with some relief. The attack begins with stupor, alternating with restlessness, increased frequency of the pulse and respiration, and a marked rise of temperature, these symptoms being due to the absorption into the blood of the products of decomposition in the digestive tracts, the presence of which was indicated by the prodromata. If not removed they continue to be absorbed, and death in a state of coma follows, sometimes attended by tonic or clonic convulsions. In consideration of the causation, Dr. Schmitz treats this condition by rapid evacuation of the intestine by means of large doses of castor oil, and he has frequently found the expulsion of large masses of black and offensive stools to be followed by a speedy disappearance of the alarming symptoms.

